# A New Way of Fixing the Kidney

**Published:** 1902-07-05

**Authors:** 


[1] Lancet, June 28.

### A NEW WAY OF FIXING THE KIDNEY.

The methods employed by different operators for fixing the kidney in the operation of nephropexy may be divided into two classes, according as they do or do not involve decortication or stripping off of the capsule. In the first class the capsule is stripped off a certain area, and then the kidney is held in position by sutures extending either through its substance or only through the reflected capsule, the intention being that the organ shall be held in place not merely by the sutures but by the broad attachment resulting from the union of the decorticated area with the tissues against which it lies. In the second class the suspension of the kidney depends primarily upon the sutures, aided by such union as may take place between those tissues, external to the capsule, which may have been torn or divided during the operation. Mr. Thomas Cawardine[1] has now endeavoured to combine the broad attachment which is gained by decortication with the advantage of not interfering with the capsule. The plan he adopts consists in freely painting the whole surface of the kidney, except the hilum with the strongest liquid carbolic acid, so that the surface becomes covered with granulation tissue within a few days. The painting is best done after the supporting sutures have been inserted, but before they are tied. In four cases, in which the kidneys were suspended by gauze, slings, and packing, he has been able to watch the surface of the kidney from ten days to three weeks afterwards, and he has observed the granulations and lymph rapidly form, followed by intimate and firm incorporation of the kidney with the surrounding tissues.

